# Hydrophobia

**Published:** 1890-02-22

**Authors:** 


### HYDROPHOBIA.

A case of hydrophobia has recently been reported from University College Hospital, under the care of Dr. Sidney Ringer. A girl, bitten by a dog in the face five weeks previously, was admitted. At the time the injury was inflicted the wounds were cauterised, and nothing extraordinary was noticed for five weeks afterwards. Then the child became dull, lost appetite, and complained of thirst, but shuddered at the sight of water, and would not try to drink it. There was also "catching" of the breath on attempt to swallow. The spasm on attempting to drink resembled the inspiratory efforts made when cold water is dashed on the body. If pressed to drink, the spasms became general, affecting the muscles of the legs. A 5 per cent. solution of cocaine as a paint for the throat, succus conii every two hours, and peptonised milk by rectum were prescribed. The next day chloroform inhalations were given; spasm on drinking was not so marked, and there was no abhorrence at sight or sound of water. Mercurials were now ordered, but in the afternoon she was at times maniacal. Chloral hydrate and bromide of potassium were given *per rectum*, and a subcutaneous injection of morphia. On the third day there was more general spasm, tending to opisthotonos, and the left side of the mouth was occasionally drawn to one side, but she took liquid nourishment with little spasm. On the fourth day there was much restlessness, incoherency, and delusions. Also conjunctivitis of the left eye. She was again put under chloroform and fed by a catheter in the œsophagus. But strength was gradually lost; the legs and arms were occasionally jerked about, and, losing consciousness, she died in the evening. Throughout the case temperature and pulse were high, and respirations from 16 to 20. A *post-mortem* showed engorgement of the spinal vessels, especially at the sides of the canal, while sections of the cord were extremely vascular, and the grey matter looked pink. Microscopical examination showed that in the cervical region of the cord the capillaries were uniformly injected with blood, most evident in the grey matter. In the lower part of the medulla the lymphatic sheaths were swollen, and in the foor of the fourth ventricle they were distended by leucocytes. General congestion was everywhere present, but no hæmorrhages were seen, and there was no obvious change in the appearance of nerves. Dr. Arkle makes some remarks on this case, to the effect that the period of incubation was rather short; that the behaviour of the dog which bit the child was characteristic of rabies; that the initial symptoms were typical; that the spasm, although present, was never very distressing; that mental disturbance was very pronounced throughout; and that the case is another instance of how little can be done remedially in hydrophobia. In our experience the symptoms of hydrophobia and the time of incubation vary greatly. In some cases, pain in the wound or numbness of the limb has been observed as a first sign, also shivering, or persistent vomiting. Aversion to drink is generally more prominent than in the present case, but sometimes the person can swallow water if the eyes are closed. In children, as a general rule, the most prominent symptom is suffocative spasm, probably because children do not feel the fear which the disease creates in adults, rendering other symptoms more obstrusive. In adults there is always from the first more or less mental distress, which eventually may proceed to maniacal frenzy. Often there is little or no febrile action, but in the present case the temperature was high throughout. Towards the end symptoms may simulate meningitis, or there may be a deceitful calm, the special symptoms remitting or disappearing. Although in most cases the main symptoms are dread of water, secretion of viscid mucus, hawking and spitting, with paroxysmal attacks of a spasmodic nature, still the variety in minor points are such as to entitle hydrophobia to be regarded as a Protean malady.

